# Determination of Artificial Sweeteners in Commercial Beverages: Do We Know What We Are Consuming?

**DOI:** 10.3390/jox15050164

**Published:** 2025-10-11

**Authors:** Mar Castellanos, Juan M. Sanchez

**Affiliations:** 1 A Coruña Biomedical Research Institute (INIBIC), 15006 A Coruña, Spain; maria.del.mar.castellanos.rodrigo@sergas.es; 2 Department of Neurology, A Coruña University Hospital, 15006 A Coruña, Spain; 3 Department of Physiotherapy, Medicine and Biomedical Sciences, A Coruña University, 15006 A Coruña, Spain; 4 Chemistry Department, University of Girona, 17003 Girona, Spain

**Keywords:** sweeteners, beverages, acesulfame K, aspartame, saccharin

## Abstract

Non-nutritive artificial sweeteners (NASs) are xenobiotics widely used in the food industry as sugar substitutes, since they provide few to no calories compared to sucrose. While NASs are considered safe at the acceptable daily intake (ADI) established by regulatory agencies, there is increasing controversy regarding their potential ability to promote metabolic derangements, especially to disrupt the gut microbiome balance. In this study, we analyzed a large cohort of the most commonly consumed beverages in Spain, categorizing them by the type of soda to determine the composition and content of the most frequently used NASs in the food industry. All commercial NAS formulations analyzed contained mixtures of different NASs. The NAS contents were always within regulated limits, although some samples yielded values close to these thresholds. Most soda samples analyzed contained NASs, even though the majority were not labeled as “zero sugars”, “no sugar added”, or “reduced calories”, which may mislead consumers. A preliminary statistical evaluation of the obtained results (cluster analysis) suggests that beverages can be grouped into three distinct clusters based on the total amount of NAS present in the samples. Differences in the total NAS content were significant among the three groups, with one cluster showing two- and four-fold higher levels than the others.

## 1. Introduction

Total sugars in foods include naturally occurring sugars, such as those present in fruits; vegetables; and dairy products, as well as added sugars, such as sucrose or dextrose, and sugars present in syrups and honey. These are often added during food preparation or processing to enhance sweetness, flavor, texture, color, and preservation.

Added sugars are only caloric sweeteners with minimal nutritional value beyond energy and may have deleterious health consequences, as a high intake of free sugars is associated with poor dietary quality and obesity (1 g of carbohydrates is considered to provide 4 kcal). The American Heart Association recommends limiting added sugars to no more than 6% of the daily caloric intake [1]. Based on the recommended daily caloric intake, ranging from 2000 to 3000 kcal for men, 1600 to 2400 kcal for women, and 1000 to 2400 kcal for children (depending on age, sex, and activity level), the suggested limits for added sugars are around 150 kcal (no more than 37 g) for men, 100 kcal (25 g) for women, and 60 to 144 kcal (15–36 g) for children. The World Health Organization has also issued guidance on reducing free sugar consumption [2]. For these reasons, the food industry has increasingly turned to non-nutritive artificial sweeteners (NASs) as alternatives to added sugars, aiming to lower calorie and sugar contents in foods and beverages without compromising sweetness.

Non-nutritive artificial sweeteners are xenobiotics with a high sweetening power (often hundreds of times greater than sucrose), so small amounts are sufficient to achieve the same sweet taste [3]. They elicit sweetness by binding to and activating sweet taste receptors in the oral cavity [4]. Although no clinical evidence indicates differential metabolic effects among NASs, their biological fates in the body differ [5,6]. For example, aspartame is rapidly broken down in the small intestine to aspartic acid (Asp), phenylalanine (Phe), and methanol, whereas other NASs, such as acesulfame K, remain intact, are absorbed across the gut, and are excreted unchanged from the mammalian body [6,7]. For this reason, most NASs are generally considered metabolically inert. However, this assumption remains debated [5], as growing evidence suggests their potential to promote metabolic disturbances, particularly through the disruption of the mammalian gut microbiome [8,9,10,11]. Furthermore, recent studies have reported associations between the consumption of NAS-containing soft drinks and increased risks of stroke and dementia [12,13,14]. It is also important to note that NASs constitute a heterogeneous group of compounds with distinct chemical structures, sweetness intensities, and metabolic pathways [15], which may result in different physiological effects in humans [16].

At present, NAS intake within the acceptable daily intake (ADI) established by regulatory agencies is considered safe. The ADI is defined as “an estimate of the amount of a food additive that can be ingested on a daily bases over a lifetime without appreciable risk to heath” [17]. The U.S. Food and Drug Administration (US-FDA) has approved six NASs as food additives: acesulfame K, aspartame, sucralose, saccharin, advantame, and neotame [18]. Neotame and advantame are chemically modified forms of aspartame with greater stability and heat resistance [19]. The European Union (EU) has approved nine NASs: the six authorized by the US-FDA plus cyclamates, neohesperidin dihydrochalcone (NHDC), and aspartame–acesulfame salt [20]. The EU list also includes steviol glycosides and thaumatin, which are classified as natural sweeteners.

Among these compounds, aspartame has generated the most recent controversy, as it was classified as a “possible carcinogenic to humans” (Group 2B) by the International Agency for Research on Cancer (IARC) [21]. Notably, despite being the only sweetener classified as a possible carcinogenic by IARC, aspartame has the highest ADI value (40 mg/kg body weight), whereas ADI values for the other NASs range from 2 mg/kg for neotame to 20 mg/kg for NHDC [22]. Another concern associated with aspartame is that it is metabolized in the gastrointestinal tract into methanol, Asp, and Phe. Phenylalanine can be harmful for individuals with phenylketonuria (PKU), a rare genetic metabolic disorder characterized by the inability to metabolize Phe, leading to its accumulation in the blood and brain, where it causes toxicity [23]. For this reason, all foods and beverages containing aspartame must clearly identify its presence on product labels. Additionally, some studies have linked aspartame consumption to headaches in a small proportion of individuals [24,25]. More recently a high intake of NASs has also been associated with faster cognitive decline and increased risks of health issues such as blood clots [26].

Other sweeteners that are controversial include saccharin, cyclamates, and sucralose. In the 1970s, some studies reported that saccharin, at high doses, was carcinogenic to the urinary bladder in rats and mice, suggesting it might also be carcinogenic in humans [27,28,29]. As a result, saccharin was banned in Canada, and in the United States products containing saccharin were required to carry a warning label stating that it could cause cancer in laboratory animals. In 1979, the IARC classified saccharin as “possibly carcinogenic to humans” (Group 2B) [30]. However, subsequent studies demonstrated that the cancer-inducing mechanism observed in rats is not relevant to humans [31]. Consequently, since 1990 saccharin has been reclassified by the IARC as “not classifiable as to its carcinogenicity to humans” (Group 3) and is now permitted in most countries [32]. In the case of cyclamate, this sweetener remains approved in the EU but was banned in the United States in the 1970s, where its use in food products continues to be prohibited [33]. For sucralose, recent studies involving pregnant women have reported that sucralose can reach breast milk, with evidence suggesting “irreversible disruptions to the development of the fetal gut microbiota during late pregnancy, as well as in neonates and infants” [34].

Non-nutritive sweeteners can be used individually or in combination with other NASs (blends). In the food industry, blends are commonly preferred [35,36] because some NASs produce undesirable side tastes (e.g., bitterness or metallic notes) at high concentrations, which can be masked by mixing different NASs [37]. Moreover, sweetener blends often have a synergistic effect, with the mixture providing greater sweetening power compared to each NAS alone [36,37]. Among these, the combination of aspartame and acesulfame K is one of the most widely used blends [5,36]. In this formulation, aspartame masks the bitter aftertaste of acesulfame K [35] and helps to better replicate the taste and texture of sucrose-sweetened products [38]. Ratios of aspartame to acesulfame K ranging from 1:1 to 4:1 are commonly used to achieve an optimal flavor profile [39].

Despite the importance of knowing the quantitative composition of NASs in foods and beverages, current regulations only require manufacturers to report the qualitative composition on product labels, with no information on the actual amounts present. Regulations generally specify only the maximum allowable levels of NAS for different categories of foods and beverages, such as Regulation No. 1333/2008 in the EU [20]. Unfortunately, most studies on sweeteners have focused primarily on developing and validating analytical methods [40,41,42], providing limited information about the concentrations of NASs in commonly consumed beverages. Moreover, some of these studies have reported that certain samples contain NAS levels exceeding regulatory limits [40]. Therefore, it is important to conduct studies that inform consumers about both the individual and total contents of NASs in commercial products. The present study evaluated a large cohort of soda beverages commonly consumed in Spain to assess the quantitative content of NASs and to identify potential trends in their use.

## 2. Materials and Methods

### 2.1. Chemicals, Reagents, and Solutions

In this study, only three NASs were quantitatively determined: acesulfame K, aspartame, and saccharin. Sucralose and cyclamate are also commonly added to soft drinks; however, these compounds lack chromophores and show poor sensitivity in the detection methods used (UV and fluorescence, FL) [43]. For cyclamate specifically, the HPLC method employed enabled qualitative detection at concentrations above 50 mg·L^−1^ but did not allow accurate quantification in most samples. Of the seventeen samples that declared cyclamate on their labels, its presence was detected in eight, but it was quantified in only two. Therefore, neither sucralose nor cyclamate were included in the statistical analyses.

Acesulfame K, aspartame, saccharin, and cyclamate (≥99.0% purity, food analysis-grade) were purchased from Sigma-Aldrich (Steinheim, Germany). Phosphoric acid (85%), potassium dihydrogen phosphate, and HPLC-grade acetonitrile (gradient grade, ≥99.9%) were also obtained from Sigma-Aldrich.

Individual stock standard solutions were prepared in ultrapure milli·Q water (Millipore Ibérica, Barcelona, Spain) and stored at 4 °C. Working solutions were prepared by appropriate dilution with Milli·Q water. The HPLC mobile phase consisted of a binary mixture of acetonitrile and 10 mM phosphate buffer (pH 2.4) [43]. The solutions of the mobile phase were degassed and filtered with 0.45 µm filters before use.

### 2.2. Samples

Forty-three types of beverages were purchased from various local stores. A preliminary study estimated the volume of refreshing beverages consumed in Spain in 2024 and found that, from a total of 1701.16 million liters consumed, 43% corresponded to cola sodas, 9% to orange sodas, 8% to Spanish “gaseosa” (a colorless, sweetened carbonated drink without added flavor), 8% to isotonic beverages, 8% to lemon sodas, 5% to tea- or coffee-based beverages, 2% to tonic waters, and the remainder to other types of refreshing beverages [44]. To reflect similar consumption percentages in the beverages analyzed, the 43 commercial products were classified as follows: 12 (27.9%) cola sodas (type A, Table 1), 8 (18.6%) orange sodas (type B), 2 (4.7%) Spanish “gaseosa” (type C), 2 (4.7%) isotonic beverages (type D), 9 (20.9%) lemon sodas (type E), 4 (9.3%) tea-based beverages (type F), 4 (9.3%) tonic waters (type G), and 2 (4.7%) energy drinks (type H).

For each beverage type, samples from different commercial brands (including store brands) were analyzed, totaling nine distinct brands. Both regular and sugar-free variants of each brand and beverage were included. For cola sodas, this included regular, non-sugar (zero), non-sugar/non-caffeine (zero zero), non-caffeine, and light versions.

To assess variability between production lots of the same beverage type, independent samples of the same product were purchased from stores in different locations, ensuring that the production lots differed.

All samples were degassed using an ultrasonic bath to remove carbon dioxide, followed by 1:2 dilution with Milli·Q water [43]. All solutions, including samples and standards, were filtered through 0.45 µm cellulose acetate syringe filters prior to HPLC analysis. For all beverages, independent duplicates were analyzed to assess reproducibility, with relative standard deviation (RSD) values below 4% in all cases.

### 2.3. Instrumentation

Chromatographic determinations were performed using an Agilent 1260 Infinity II HPLC system (Agilent Technologies, Santa Clara, CA, USA), equipped with a UV diode array detector (1260 DAD HS, Agilent Technoloogies) and a fluorescence detector (1260 FLD, Agilent Technologies). A 20 µL injection volume was delivered via an autosampler (1260 Vialsampler, Agilent Technologies).

Separations were carried out using a binary gradient elution system (Table 2) [43] on a 20 cm × 0.46 cm i.d. column, packed with 5 µm Kromasil 100-5-C18 silica (Teknokroma, Barcelona, Spain). The mobile phase flow rate was set at 1.0 mL·min^−1^, and all chromatographic runs were conducted at 25 ± 0.5 °C.

UV detection wavelengths were set as follows: 227 nm for acesulfame K, 205 nm for saccharin and aspartame, and 195 nm for cyclamate. Additionally, saccharin was also monitored by FL detection, with an excitation wavelength of 250 nm and an emission wavelength of 440 nm.

### 2.4. Statistical Analysis

Statistical analyses were performed using SPSS for Windows, version 29.0.1.0 (SPSS Inc., Chicago, IL, USA). A significance level of *p* < 0.05 was considered statistically significant. Preliminary assessment of data distribution using the Shapiro–Wilk test indicated non-normal distributions for all sweeteners (*p* < 0.007). When samples were categorized as added sugar versus non-added sugar, non-normal distributions were observed for all analytes (*p* < 0.003), except for acesulfame K in the non-added sugar group (*p* = 0.320). Therefore, non-parametric statistic tests were applied for comparisons where appropriate.

For assessment of differences between beverage types, a parametric ANOVA test was applied because some sample types had n = 2, which precluded the use of non-parametric tests.

## 3. Results

A total of 43 commercial beverages were analyzed (Table S1 in the Supplementary Materials presents the results obtained for each sample and all target analytes). Of these, 24 beverages (56%) were labeled as “no added sugars”, while 19 (44%) were labeled as “added sugars”. Overall, at least one of the targeted NASs was detected in 39 samples (91%). This indicates that many beverages labeled as “added sugars” also contained NAS. Only four samples (9%), all labeled as “added sugars”, showed no presence of NAS. These findings are consistent with the qualitative information reported on their labels. Table 1 presents the descriptive statistics obtained for each of the three quantified sweeteners, with results separated by beverage type.

**Figure 1:**
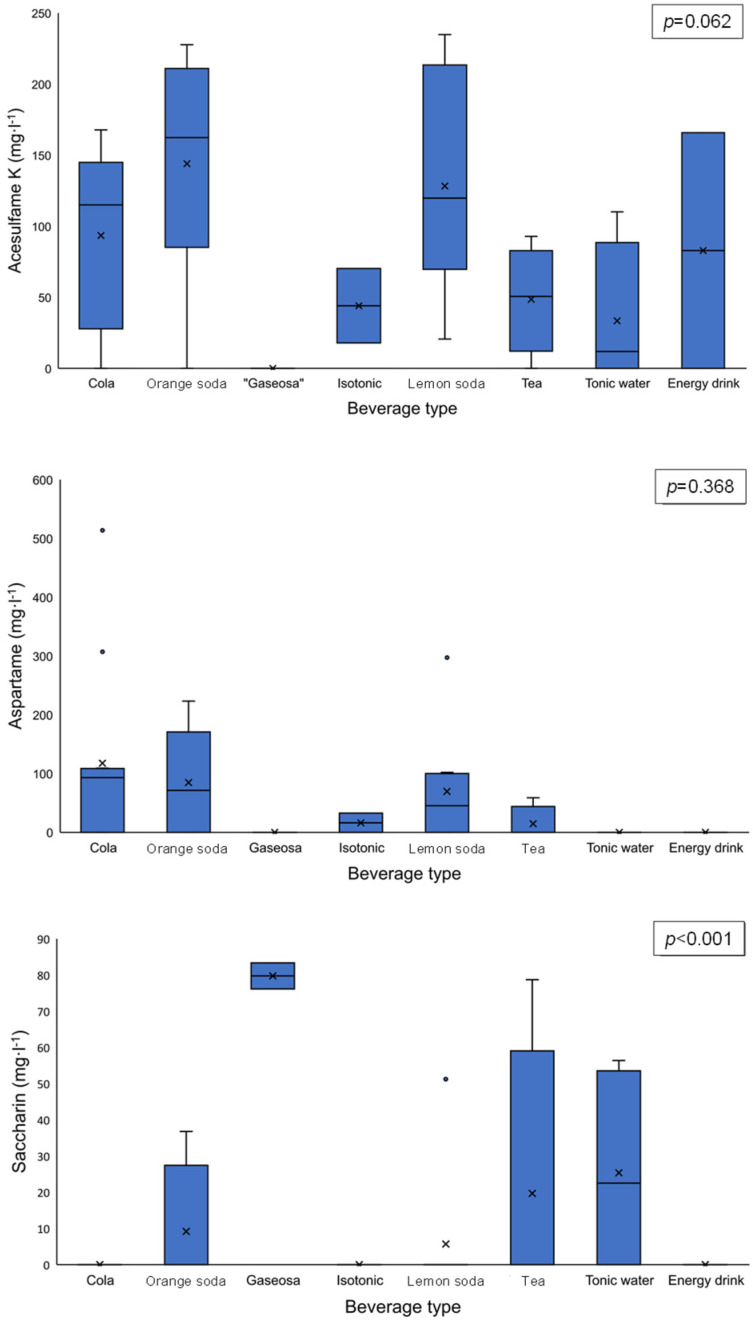
Box plots of acesulfame K, aspartame, and saccharin concentrations. An ANOVA parametric test was applied, with n = 2 for Spanish “gaseosas”, isotonic beverages, and energy drinks (see Table 1).

The ANOVA of the results showed no significant differences in the acesulfame K (*p* = 0.062) or aspartame (*p* = 0.368) content between beverage types. Only saccharin showed a significant difference (*p* < 0.001) (Figure 1). The Tukey post hoc test revealed that only Spanish “gaseosa” exhibited significant differences in saccharin contents.

A blend of NASs was used in the 39 samples where sweeteners were detected. The most common combination was aspartame/acesulfame K, found in 8 samples (18.6%). This mixture also appeared in 14 additional samples that contained three or four NASs in their blends. The second most frequent combinations were acesulfame K/sucralose and acesulfame K/aspartame/cyclamate, each found in seven samples (16.3%).

**Figure 2:**
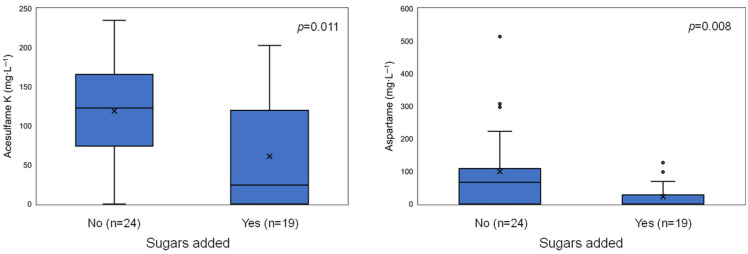
Box plots of acesulfame K and aspartame concentrations comparing “no sugars added” and “sugars added” beverages. Statistical analysis was performed using the non-parametric Mann–Whitney U test.

When the distribution of each analyte was compared between the group of “added sugars” and “no added sugars” (Figure 2), significant differences were observed for acesulfame K (*p* = 0.011) and aspartame (*p* = 0.008). Saccharin did not show a significant difference (*p* = 0.052); however, it was detected in only one of the nineteen “added sugars” samples (a tonic water, 5%).

All beverages labeled as “no added sugars” (n = 24) were found to contain NAS. In 20 of these samples (83%), a mixture of NASs was detected, with the aspartame/acesulfame K combination being the most common (Table S1 in the Supplementary Materials). In the remaining four samples (17%), only one of the targeted sweeteners was detected; however, their labels also declared the presence of other non-analyzed sweeteners, such as sucralose, stevia glycosides, and NDHC. Thus, all beverages labeled as “no added sugars” contained a mixture of at least two different NASs.

Of the 19 beverages labeled as “added sugars”, NASs were detected in 15 samples (79%), with acesulfame K being the most prevalent (n = 13). Only four “added sugar” samples (21%) contained no NAS: two cola sodas (including one store brand), one tonic water, and one energy drink.

As shown in Figure 2, three of the “no added sugars” samples exhibited aspartame levels that appeared as extreme values in the box plot. To determine whether these results were outliers due to systematic analytical errors or valid measurements, five independent samples of different commercial sodas (including the three identified as extreme values in Figure 2) were analyzed. Samples were obtained from different stores, and care was taken to ensure that production lots differed to guarantee independence. The results confirmed that these extreme values were neither outliers nor analytical errors (Table 3). Furthermore, the precision achieved in all analyses (RSD < 5%) suggests that the amounts of NAS added by producers are fairly consistent across production lots.

**Figure 3:**
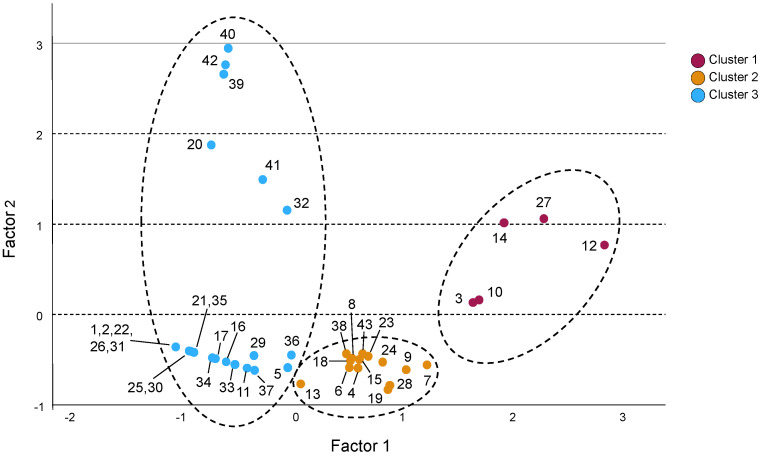
Factor analysis of mixed data. Samples in cluster 1 are shown in red, those in cluster 2 are shown in brown, and those in cluster 3 are shown in blue. Numbers correspond to sample numbers in Table S1 (Supplementary Materials).

A hierarchical cluster analysis (Ward’s method) was performed to investigate potential structures within the dataset (Figure S1 in the Supplementary Materials). This analysis revealed three distinct clusters. Subsequently, a factorial analysis was conducted (Figure 3), yielding statistically significant results (*p* < 0.05) and a Kaiser–Meyer–Olkin (KMO) value of 0.57.

## 4. Discussion

In this study, NASs were analyzed in 43 different commercial beverages: 44% of the samples were labeled as containing added sugars, whereas 56% were beverages without added sugars. Despite the fact that 44% of the analyzed beverages contained added sugars, NASs were detected in 91% of all samples. Among the nineteen samples with added sugars, NASs were absent in only four beverages (21%) and detected in the remaining fifteen (79%). Some of the beverages with added sugars (n = 8, 42%) were explicitly labeled as “reduced-calorie” or “low-calorie”. The use of NAS in these reduced-calorie beverages has also been reported in recent studies analyzing sodas in other European countries [45,46,47]. This practice reflects the food industry’s commitment to reducing sugar and caloric contents in soft drinks while preserving sweetness. Accordingly, the term “reduced sugar” typically refers to foods containing at least 25% less sugar than the reference amount [48].

Seven of the analyzed beverages with added sugars, in which NASs were detected, did not provide any specific information about calorie reduction on the principal field of their labels, as required by EU regulations. In these samples, the presence of the NAS was only disclosed in the list of ingredients on the back part of the packaging. Similar labeling inconsistences have been reported by Knezovic et al. [47]. These seven beverages included products from an international company and a store brand. A decade ago the international company announced that by 2025 at least two-thirds of its drinks would have reduced calorie contents in response to consumer preferences [49]. NASs were detected in all beverages analyzed from this company, both with and without added sugars, and the caloric content of sugar-containing beverages was reduced through the use of NASs. For example, its regular cola did not contain NAS in Spain until 2023, when it was labeled with a caloric content of 43 kcal/100 mL. The reformulated version now contains acesulfame K (97 mg·L^−1^ detected) and sucralose, reducing its caloric content to 18 kcal/100 mL (58% reduction).

Overall, NASs were detected in 39 of the beverages analyzed (91%), consistent with findings from other studies on soft drinks (e.g., Silva et al. [45] reported NAS in 85% of samples and Knezovic et al. [47] in 76%). The most frequently quantified sweetener was acesulfame K, detected in 33 samples (79%), followed by aspartame in 22 beverages (51%) and saccharin in 8 samples (19%). Cyclamate was detected but not quantified in 8 samples, although label information indicated its presence in 17 samples (40%). Sucralose was not determined analytically in this study, but the label information showed it was present in 13 samples (30%).

The proportion of beverages containing acesulfame K in this study (79%) was comparable to values reported elsewhere (76% in [46], 74% in [50], 62% in [47], and 60% in [40]). Sezgin et al. [41] reported a higher proportion (90%), but their study involved only a small number of beverages, all formulated with NAS, to validate a new analytical method.

In contrast, the proportion of beverages containing aspartame (51%) was lower than in some previous reports (73% in [46] and 82% in [50]), though it was closer to the results of a study on Spanish beverages (32% [40]). These discrepancies may be partly explained by the timing of the sample collection: earlier studies analyzed beverages produced before 2023, the year in which the IARC classified aspartame as “possibly carcinogenic”, whereas all samples in the present study were manufactured in 2024 or 2025. This interpretation is supported by a recent study from Croatia, which found aspartame in only 32% of analyzed beverages [47].

It is noteworthy that among the seven sugar-containing samples that did not report any calorie reduction information on their labels but contained NAS aspartame was detected in only two samples (both from a store brand). Aspartame was absent in the five samples from the international company that introduced sweeteners into these products in Spain in 2023. In contrast, acesulfame K was detected in all seven samples.

A preliminary hypothesis in our study was that smaller amounts of NAS might be present in fruit-flavored sodas due to the natural presence of fructose in these beverages (juice contents ranged from 1 to 10% in these flavored sodas, types B and E in Table 1). This hypothesis appeared to be supported by two studies analyzing sodas in Portugal, which found that cola sodas had the highest contents of aspartame and acesulfame K [45,50]. However, when all analyzed samples were compared by beverage type, no significant differences were observed for acesulfame K and aspartame (Figure 1). On the contrary, the highest concentrations of acesulfame K were consistently found in flavored sodas.

The use of saccharin has declined in recent years. This sweetener was detected in only 19% of the samples, consistent with other recent studies reporting its presence at <20% [40,41]. The saccharin content was significantly higher in Spanish “gaseosa” compared with other beverage types (Figure 1). Notably, the maximum permitted concentration of saccharin in sodas in the EU is 80 mg·L^−1^, except for Spanish “gaseosa”, where the limit is set at 100 mg·L^−1^ [20]. The two “gaseosa” samples analyzed contained 76 and 83 mg·L^−1^, concentrations that comply with the legal limit for this beverage but would exceed, or be at, the limit for other sodas. It should also be noted that all commercial Spanish “gaseosa” currently on the market are sugar-free; therefore, NASs are added to reproduce the sweetness of the original versions, with saccharin and cyclamate being the most commonly used NASs in this beverage type.

It is also important to note that all samples in which NASs were detected contained a blend of sweeteners. Although only one NAS was quantified in 15 samples, their labels indicated the presence of additional NASs that were not analyzed in this study, such as sucralose, steviol glicosides, NHDC, and cyclamate. As reported in the Section 3, the most commonly found mixture was aspartame/acesulfame K (in 8 samples, 18.6%), with this combination also present in more complex blends in 14 additional samples. This mixture has also been reported as the most common blend found in other studies from Spain [40] and Germany [46]. However, blends appear to vary by country; for example, a recent study in Croatia found sucralose/acesulfame K to be the most prominent combination [47]. Interestingly, although aspartame/acesulfame K ratios ranging from 1:1 to 4:1 have been reported as optimal for flavor profiles [39], only 3 of the 22 samples containing this mixture fell within this range (1:1, 3.4:1, and 3.5:1). Two samples exhibited much higher ratios (13.1:1 and 14.4:1), while seventeen samples (77.3%) had ratios below 1:1 (ranging from 0.2:1 to 0.8:1). This suggests a trend of using higher levels of acesulfame K relative to aspartame, which may be associated with the recent classification of aspartame as a possible carcinogen.

When the samples were grouped into sugar-containing and sugar-free categories, significant differences were observed for aspartame and acesulfame K (Figure 2). For saccharin, most samples showed no detectable levels, rendering the statistical analysis inconclusive. These results support the hypothesis that in “reduced-calorie” beverages with added sugars, smaller amounts of NAS are required to achieve an equivalent sweetness compared to sugar-free beverages, as a significant portion of the sweetness is still provided by the added sugars.

Only four samples (two cola sodas, one tonic water, and one energy drink) classified as sugar-containing beverages did not include NAS. The amount of added sugars in these samples ranged from 7.8 to 11 g/100 mL (mean = 9.9, SD = 1.5). The remaining 15 samples in the sugar-containing group were reduced-calorie beverages, in which added sugars had been partially replaced with NAS. According to their labels, the sugar content in this subgroup ranged from 2.4 to 7 g/100 mL (mean = 4.6, SD = 0.9). This difference in the added sugar content between the two subgroups was statistically significant (*p* < 0.001), corresponding to a mean reduction of 54%.

The most interesting trend emerged from the hierarchical cluster analysis (Figure S1 in the Supplementary Materials), which showed that the samples can be grouped into three clusters according to their total NAS content. Figure 3 presents the distribution diagram of the samples after applying the factor analysis.

Cluster 1 included samples with the highest concentrations of aspartame (mean = 305 mg·L^−1^), intermediate levels of acesulfame K (mean = 131 mg·L^−1^), detectable saccharin (mean = 15 mg·L^−1^), and no added sugar. The mean total NAS concentration in cluster 1 was the highest, reaching 451 mg·L^−1^. This cluster contained only five samples, all labeled as “zero” or “light” sodas. Notably, although this study analyzed beverages from nine different international, national, and store brands, all five samples in cluster 1 belonged to just two of the most prominent international soda companies.

Cluster 2 included 14 samples, characterized by the highest concentrations of acesulfame K (mean = 168 mg·L^−1^), intermediate levels of aspartame (mean = 83 mg·L^−1^), the absence of saccharin, and reduced amounts of added sugars (mean = 1.4 g/100 mL). Notably, 10 of the samples in this cluster (71%) contained no added sugars. The mean total NAS concentration in cluster 2 was 251 mg·L^−1^, approximately half of that observed in cluster 1.

Cluster 3 grouped 24 samples with the lowest concentrations of acesulfame K (mean = 42 mg·L^−1^) and aspartame (mean = 5 mg·L^−1^) but detectable levels of saccharin (mean = 16 mg·L^−1^). The mean total NAS concentration in this cluster was 63 mg·L^−1^, corresponding to about 25% of the mean in cluster 2 and 14% of that in cluster 1. Although cluster 3 included most of the sugar-containing sodas (mean = 4 g), eight samples (33%) in this cluster had no added sugars.

Finally, it should be noted that all analyzed samples contained individual NAS levels below the maximum limits established by the European legislation for sodas. However, the results obtained suggest that those sodas in cluster 1, with a mean total NAS concentration of 451 mg·L^−1^, may be of the greatest concern due to their high overall NAS content, despite containing no added sugars.

## 5. Conclusions

Consumers often presume that sweeteners are only used as substitutes for added sugars in beverages marketed as sugar-free. The results of this study, however, show that 91% of the beverages analyzed contained NAS, even though 44% of the samples also included added sugars. In fact, 79% of the sugar-containing beverages also contained NAS.

The most significant finding of this study is that the analyzed sodas can be grouped into three distinct clusters, differentiated by their total NAS content. Large differences were observed between clusters: cluster 1 had the highest mean NAS concentration (451 mg·L^−1^), cluster 2 contained approximately half that amount (251 mg·L^−1^), and cluster 3 showed the lowest levels (63 mg·L^−1^). These results suggest that beverages in cluster 1 may pose the greatest concern due to their elevated NAS content. It should be noted that a recent study [26] associated a higher total NAS intake with faster cognitive decline, defining high consumption as >191 mg NAS/day. In an eight-year prospective study, the authors concluded that the memory and thinking decline was 62% faster, and verbal frequency decline was 173% faster, among individuals consuming high amounts of NAS. Moreover, there was little difference among individual NAS types, providing no evidence that one NAS was more detrimental than another. This suggests that total NAS intake contributes cumulatively to the risk. Therefore, the extended use of NAS blends by the beverage industry may further increase potential health risks. As found in another study [47], although the use of blends can enhance flavor and produce synergistic effects, it also allows for the inclusion of higher total amounts of NAS in food products, since current legislation sets maximum limits for each individual NAS but does not regulate their combined concentration.

## Figures and Tables

**Table 1:** Descriptive statistics for the sweeteners analyzed in this study. Samples are grouped by beverage type: A: cola sodas; B: orange sodas; C: Spanish “gaseosa”; D: isotonic drinks; E: lemon sodas; F: tea beverages; G: tonic waters; and H: energy drinks. Values are reported in mg·L^−1^. For calculations, a value of 0 was assigned when the analyte was not detected in a sample (nd). Q1: 25% percentile, Q2: 50% percentile (median), and Q3: 75% percentile.

Sweetener	Drink Type (n, m) ^1^	Mean	SD	Q1	Median (Q2)	Q3	Min	Max
Acesulfame K	A (12, 10)	94	62	28	115	145	nd	168
	B (8, 7)	144	78	85	162	211	nd	228
	C (2, 0)	-	-	-	-	-	-	-
	D (2, 2)	44	37	-	44	-	18	70
	E (9, 9)	128	76	70	120	120	21	235
	F (4, 3)	49	38	12	51	83	nd	93
	G (4, 2)	33	52	nd	12	88	nd	110
	H (2, 1)	83	117	-	83	-	nd	166
Aspartame	A (12, 8)	118	151	nd	93	109	nd	514
	B (8, 5)	85	87	nd	71	171	nd	223
	C (2, 0)	-	-	-	-	-	-	-
	D (2, 1)	16	23	-	16	-	nd	33
	E (9, 6)	70	94	nd	45	100	nd	297
	F (4, 1)	15	29	nd	nd	44	nd	59
	G (4, 0)	-	-	-	-	-	-	-
	H (2, 0)	-	-	-	-	-	-	-
Saccharin	A (12, 0)	-	-	-	-	-	-	-
	B (8, 2)	9	17	nd	nd	nd	nd	37
	C (2, 2)	80	5	-	80	-	76	83
	D (2, 0)	-	-	-	-	-	-	-
	E (9, 1)	6	17	nd	nd	nd	nd	51
	F (4, 1)	20	39	nd	nd	59	nd	78
	G (4, 1)	25	30	nd	23	53	nd	56
	H (2, 0)	-	-	-	-	-	-	-

^1^ n is the number of samples analyzed from the assessed type, and m is the number of samples of this type where the sweetener was detected.

**Table 2:** HPLC binary gradient applied ^1^.

Time (min)	10 mM Phosphate Buffer pH = 2.4, %	Acetonitrile, %
0	90	10
5	90	10
15	60	40
19	25	75
30	25	75
33	90	10
37	90	10

^1^ Separation of NAS was achieved within 14 min (retention times: acesulfame K = 4.51 ± 0.09 min, saccharin = 7.24 ± 0.17 min, cyclamate = 11.10 ± 0.07 min, and aspartame = 12.59 ± 0.05 min). However, the total analysis exceeded 14 min because (i) the applied method also enabled baseline separation and identification of caffeine (10.65 ± 0.02 min, present in cola samples and energy drinks) and sorbate (16.6 ± 0.10 min, a common preservative [E-202] used in many soft drinks to inhibit the growth of molds, yeasts, and bacteria) and (ii) additional time was required for proper cleaning and conditioning of the column between runs.

**Table 3:** Concentrations of sweeteners (mg·L^−1^) in beverages analyzed to assess the lot-to-lot variability (four independent samples per beverage). All samples were labeled as “zero” or “light”, with no sugars added (nd: not detected).

Sample		Acesulfame K	Aspartame	Saccharin
Cola soda #1	Mean (SD)	38.8 (0.4)	546.2 (23.0)	nd
	RSD, %	1.1	4.3	
Cola soda #2	Mean (SD)	140.8 (2.1)	99.9 (4.6)	nd
	RSD, %	1.5	4.6	
Cola soda #3	Mean (SD)	61.9 (1.9)	307.3 (6.2)	nd
	RSD, %	3.1	2.0	
Flavored soda #1	Mean (SD)	88.2 (2.4)	297.3 (4.8)	nd
	RSD, %	2.7	1.6	
Flavored soda #2	Mean (SD)	226.6 (1.2)	219.3 (8.6)	36.8 (0.3)
	RSD, %	0.6	3.9	0.7

## Data Availability

The original contributions presented in this study are included in the article/Supplementary Material. Further inquiries can be directed to the corresponding author.

## Supplementary Materials

The following supporting information can be downloaded at https://www.mdpi.com/article/10.3390/jox15050164/s1: Table S1: Results obtained in the quantification of acesulfame K, aspartame, and saccharin in the beverages analyzed; Figure S1: Dendrogram illustrating the clustering of the beverages analyzed.

## Abbreviations

The following abbreviations are used in this manuscript:

ADI	Acceptable Daily Intake
Asp	Aspartic Acid
EU	European Union
FDA	Food and Drug Administration
FL	Fluorescence
HPLC	High-Performance Liquid Chromatography
IARC	International Agency for Research on Cancer
NAS	Non-Nutritive Artificial Sweetener
NHDC	Neohesperidin Dihydrochalcone
Phe	Phenylalanine
PKU	Phenylketonuria
RSD	Relative Standard Deviation
SD	Standard Deviation
UV	Ultraviolet

## References

[1] American Heart Association Added Sugars. https://www.heart.org/en/healthy-living/healthy-eating/eat-smart/sugar/added-sugars.

[2] World Health Organization (WHO) (2015). Guideline: Sugars Intake for Adults and Children.

[3] WHO (2023). Use of Non-Sugar Sweeteners: WHO Guideline Summary.

[4] Ahmad R., Dalziel J.E. (2020). G protein-coupled receptors in taste physiology and pharmacology. Front Pharmacol..

[5] Mehat K., Chen Y., Corpe C.P. (2022). The combined effects of aspartame and acesulfame-K blends on appetite: A systematic review and met-analysis of randomized clinical trials. Adv. Nutr..

[6] Glendinning J.I. (2018). Oral post-oral actions of low-calorie sweeteners: A tale of contradictions and controversies. Obesity.

[7] Hubrecht I., Baenas N., Sina C., Wagner A.E. (2022). Effects of non-caloric artificial sweeteners on naïve and dextran sodium sulfate-exposed Drosophila melanogaster. Food Front..

[8] Suez J., Korem R., Zeevi D., Zilberman-Schapira G., Thaiss C.A., Maza O., Israeli D., Zmora N., Gilad S., Weinberger A. (2014). Artificial sweeteners induce glucose intolerance by altering the gut microbiota. Nature.

[9] Suez J., Korem T., Zilberman-Schapira G., Segal E., Elinav E. (2015). Non-caloric artificial sweeteners and the microbiome: Findings and challenges. Gut Microbes.

[10] Harrington V., Lau L., Crits-Cristoph A., Suez J. (2022). Interactions of non-nutritive artificial sweeteners with the microbiomes in metabolic syndrome. Immunometabolism.

[11] Hetta H.F., Sirag N., Elfadil H., Salama A., Aljadrawi S.F., Alfaifi A.J., Alwabisi A.N., AbuAlhasan B.M., Alanazi L.S., Aljohani Y.A. (2025). Artificial sweeteners: A double-edged sword for gut microbiome. Diseases.

[12] Pase M.P., Himali J.J., Beiser A.S., Aparicio H.J., Satizabal C.L., Vasan R.S., Seshadri S., Jacques P.F. (2017). Sugar- and artificially sweetened beverages and the risks of incident stroke and dementia: A prospective cohort study. Stroke.

[13] Debras C., Chazelas E., Sellem L., Porcher R., Druesne-Pecollo N., Esseddik Y., de Edelenyi F.S., Agaëssse C., De Sa A., Ltuchia R. (2022). Artificial sweeteners and risk of cardiovascular diseases: Results from the prospective NutriNet-Santé cohort. BMJ.

[14] Wu W., Sui W., Chen S., Guo Z., Jing X., Wang X., Wang Q., Yu X., Xiong W., Ji J. (2025). Sweetener aspartame aggravates atherosclerosis through insulin-triggered inflammation. Cell Metab..

[15] Magnuson B.A., Carakostas M.C., Moore N.H., Poulos S.P., Renwick A.G. (2016). Biological fate of low-calorie sweeteners. Nutr. Rev..

[16] Hunter S.R., Reister E.J., Cheon E., Mattes R.D. (2019). Low calorie sweeteners differ in their physiological effects in humans. Nutrients.

[17] European Food Information Council (EUFIC) (2021). What Is an Acceptable Daily Intake (ADI)?. https://www.eufic.org/en/understanding-science/article/qas-on-acceptable-daily-intakes-adis.

[18] Aspartame and Other Sweeteners in Food. US-FDA. https://www.fda.gov/food/food-additives-petitions/aspartame-and-other-sweeteners-food.

[19] Farag M.A., Rezk M.M., Elashal M.H., El-Araby M., Khalifa S.A.M., El-Seedi H.R. (2022). An updated mulfaceted overview of sweet proteins and dipeptides as sugar substitutes; the chemistry, health benefits, gur interactions, and safety. Food Res. Int..

[20] The European Parliament and the Council of the European Union (2008). Regulation (EC) No 1333/2008 of the European Parliament and of the Council of 16 December 2008 on Food Additives, Annex II, Part E.

[21] International Agency for Research on Cancer (IARC) (2014). Aspartame, Methyleugenol, and Isoeugenol (IARC Monographs on the Identification of Carcinogenic Hazards to Humans).

[22] Acceptable Daily Intake of Sweeteners in the EU. https://knowledge4policy.ec.europa.eu/health-promotion-knowledge-gateway/sugars-sweeteners-7_en.

[23] Maler V., Goetz V., Tardieu M., El Khalil A., Alili J.M., Meunier P., Maillot F., Labarthe F. (2023). Aspartame and phehylketonuria: An analysis of the daily phenylalanine intake of aspartame-containing drugs markected in France. Orphanet J. Rare Dis..

[24] Lipton R.B., Newman L.C., Cohen J.S., Solomon S. (1989). Aspartame as dietary trigger of headache. Headache.

[25] Van den Eeden S.K., Koepsell T.D., Longsteth W.T., van Belle G., Daling J.R., McKnight B. (1994). Aspartame ingestion and headaches: A randomized crossover trial. Neurologyy.

[26] Gonçalves N.G., Martinez-Steele E., Lotufo P.A., Bensenor I., Goulart A.C., Barreto S.M., Giatti L., Perim de Faria C., Bisi-Molina M.C., Caramelli P. (2025). Association between consumption of low- and non-calorie artificial sweetener and cognitive decline: An 8-year prospective study. Neurology.

[27] Reuber M.D. (1978). Carcinogenicity of saccharin. Environ. Health Perspect..

[28] Taylor J.M., Weinberger G.M., Friedman L. (1980). Chronic toxicity and carcinogenity ot the urinary bladder of sodium saccharin in the in utero-exposed rat. Toxicol. Appl. Pharm..

[29] Squire R.A. (1985). Histopathological evaluation of rat urinary bladders from the IRDC two-generation bioassay of sodium saccharin. Food Chem. Toxicol..

[30] IARC (1980). Some Non-Nutritive Sweetening Agents, IARC Monographs on the Evaluation of the Carcinogenic Risk of Chemicals to Humans.

[31] Weihrauch M.R., Diehl V. (2004). Artificial sweeteners—Do they bear a carcinogenic risk?. Ann. Oncol..

[32] EFSA Panel on Food Additives and Flavourings (FAF) (2024). Re-evaluation of saccharin and its sodium, potassium and calcium salts (E954) as food additives. EFSA J..

[33] Clemens R., Pressman P., Wallace Hayes A., Haugabrooks E., Wallace Hayes P. (2023). Chapter 4—Food additives toxicology. History of Food and Nutrition Toxicology (History of Toxicology and Environmental Health).

[34] Tkach V.V., Morozova T.V., de O’Neill Mascarenhas I., de Gomes Miranda N., Ivanushko Y.G., de Ferrao Paiva J.I., Novo Barros A. (2025). Sucralose: A review of environmental, oxidative and genomic stress. Nutrients.

[35] Zygler A., Wasik A., Namiesnik J. (2009). Analytical methodologies for determination of artificial sweeteners in foddstuffs. TrAC Trends Anal. Chem..

[36] Wang C., Liu Y., Zhao X., Liu B. (2022). Current advances and future aspects of sweetener synergy: Properties, evaluation methods and molecular mechanisms. Appl. Sci..

[37] Tan V.W.K., Wee M.S.M., Tomic O., Forde C.G. (2019). Temporal sweetness and side tastes of 16 sweeteners using temporal check-all-that-apply. Food Res. Int..

[38] Margolskee R.F., Dyer J., Kokrashvili Z., Salmon K.S.H., Ilegems E., Daly K., Maillet E.L., Nimomiva Y., Mosinger B., Shirazi-Beechey S.P. (2007). T1R3 and gustducin in gut sense sugars to regulate expression of Na^+^-glucose cotransporter 1. Proc. Natl. Acad. Sci. USA.

[39] Baron R.F., Hanger L.Y. (1998). Using acid level, acesulfame potassium/aspartame blend ratio and flavor type to determine optimum flavor profiles of fruit flavored beverages. J. Sens. Stud..

[40] Ordoñez E.Y., Rodil R., Quintana J.B., Cela R. (2015). Determination of artificial sweeteners in beverages with green mobile phases and high temperature liquid chromatography-tandem mass spectrometry. Food Chem..

[41] Sezgin B., Arli G., Öncü-Can N. (2021). Simultaneous HPLC-DAD determination of seven intense sweeteners in foodstuffs and pharmaceuticals using a core-shell particle column. J. Food Compos. Anal..

[42] Jankulovska M.S., Josimovska T., Velkoska-Markovska L. (2025). Development and validation of RP-HPLC method with UV-DAD detection for simultaneous determination of acesulfame K, sodium saccharin and aspartame in beverages. Acta Chromatogr..

[43] Hernández N., Sanchez J.M. (2025). Validation of a method for the determination of artificial sweeteners and caffeine in soft drinks: The impact of regression function selection on quantification limits considering trueness and precision. Separations.

[44] Volumen de Bebidas Refrescantes y Gaseosa Consumidas por los Hogares en España en 2023, por tipo. https://es.statista.com/estadisticas/488967/consumo-de-refrescos-y-gaseosa-en-espana-por-tipo/.

[45] Silva P.D., Cruz R., Casal S. (2021). Sugars and artificial sweeteners in soft drinks: A decade of evolution in Portugal. Food Control.

[46] Bundesinstitut für Risikobewertung (2023). Sugar alternatives: How much sweetener is there in soft drinks?: Opinion no. 006/2023 from 7 February 2023. BfR-Stellunghahmen.

[47] Knezovic Z., Jurcevic Zidar B., Pribisalic A., Luetic S., Jurcic K., Knezovic N.M., Sutlovic D. (2025). Artificial sweeteners in food products: Concentration analysis, label practices, and cumulative intake assessment in Croatia. Nutrients.

[48] McCain H.R., Kaliappan S., Drake M.A. (2018). Invited review: Sugar reduction in dairy products. J. Dairy Sci..

[49] PepsiCo to Reduce Soda Calories by 2025. https://www.convenience.org/Archive/News/NACSDailyArticles/2016/ND1018162.

[50] Lino C.M., Costa I.M., Pena A., Ferreira R., Cardoso S.M. (2008). Estimated intake of the sweeteners, acesulfame-K and aspartame, from soft drinks, soft drinks bases on mineral waters and nectars for a group of Portuguese teenage students. Food Addit. Contam. A.

