# Supplementary material for: Determination of Artificial Sweeteners in Commercial Beverages: Do We Know What We Are Consuming?
Source: J Xenobiot. 2025 Oct 11;15(5):164. doi: 10.3390/jox15050164 (PMC12565320; doi:10.3390/jox15050164)
Supplement: Supplementary file 1 [file jox-15-00164-s001.zip › jox-3903257-supplementary.pdf]

Supplementary Materials

Determination of Artificial Sweeteners in Commercial Beverages: Do We Know What We Are Consuming?

Mar Castellanos [1,2,3] and Juan M. Sanchez [1,4,*]

[1] A Coruña Biomedical Research Institute (INIBIC), 15006-A Coruña (Spain)
[2] Department of Neurology, A Coruña University Hospital, 15006-A Coruña (Spain); maria_del_mar_castellanos_rodrigo@sergas.es
[3] Department of Physiotherapy, Medicine and Biomedical Sciences, A Coruña University, 15006-A Coruña (Spain)
[4] Chemistry Department, University of Girona, 17003-Girona (Spain); juanma.sanchez@udg.edu
* Correspondence: juanma.sanchez@udg.edu; Tel.: (+34) 636569984

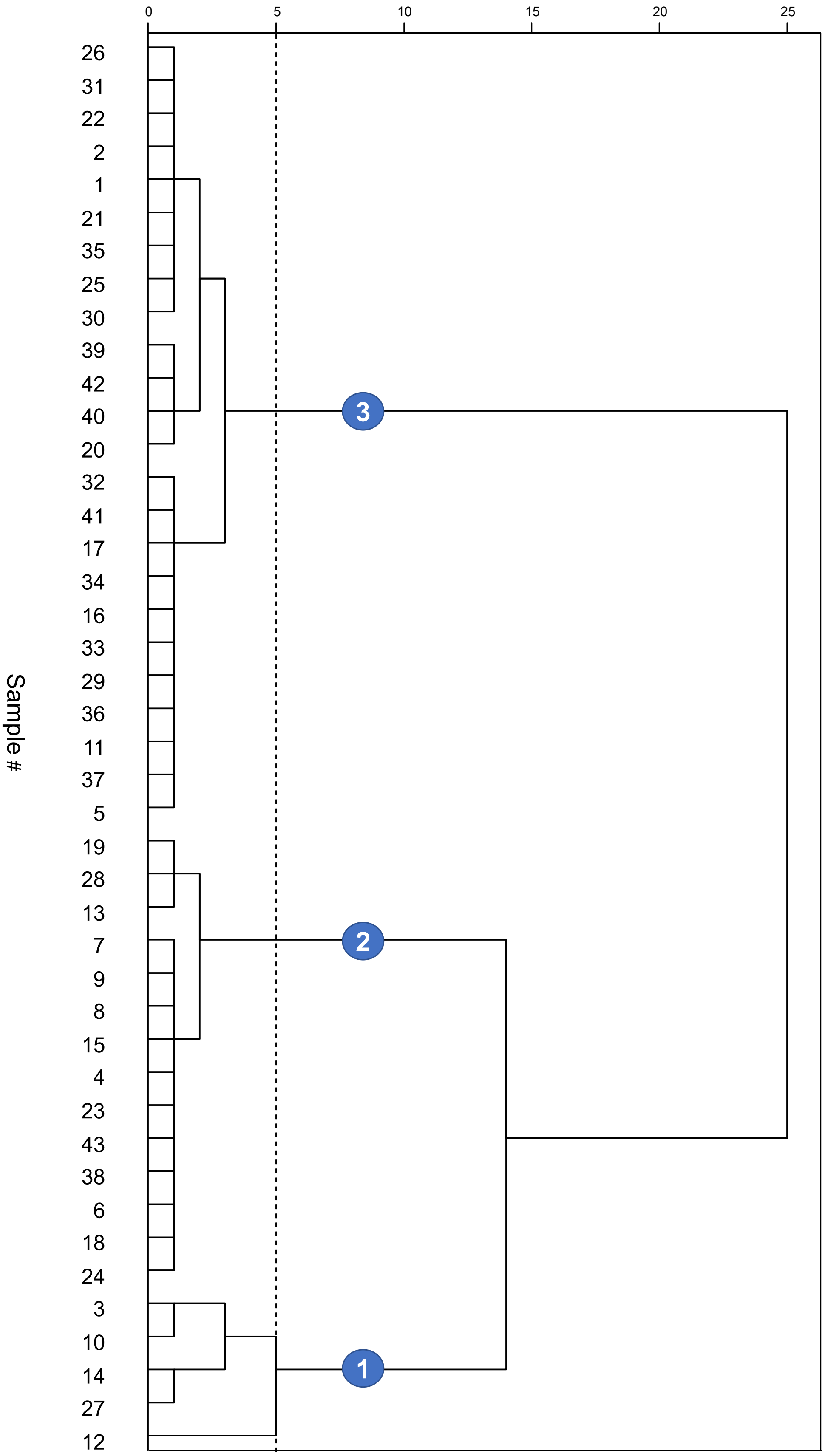

Figure S1. Dendrogram illustrating the clustering of the beverages analyzed in this study. The numbers inside the blue circle names the three cluster designed.

Table S1. Results obtained in the quantification of acesulfame K, aspartame and saccharin in the beverages analyzed. Concentrations reported in mg·L^{-1} (d: detected; --: non-detected).

[1]As labeled by the manufacturer; E numbers: E950-Acesulfame K; E951-Aspartame; E952-Cyclamates; E954-Saccharins; E955-Sucralose; E959-NHDC; 960-Steviol glycosides.

Sample #	Type	Store brand	Sweeteners added (E numbers) [1]	Sugars added (g/100 mL)	Acesulfame K (SD)	Aspartame (SD)	Saccharin (SD)	Cyclamates
1	A: cola	No	**None**	Yes (10.6)	--	--	--	--
2	A: cola	Yes	**None**	Yes (10.4)	--	--	--	--
3	E: lemon soda	No	950, 951	No	88,1 (0.6)	297.3 (2.4)	--	--
4	A: cola	No	950, 951, 952	No	144.2 (0.7)	84.9 (0.3)	--	--
5	E: lemon soda	Yes	950, 951, 952	Yes (4.6)	119.8 (5.1)	27.9 (0.9)	--	d
6	B: orange soda	Yes	950, 951	Yes (4.8)	159.1 (0.6)	69.1 (0,2)	--	d
7	B: orange soda	No	950, 951, 959	Yes (4.5)	202.8 (4.7)	126.8 (3.0)	--	--
8	A: cola	No	950, 951, 952, 960	No	138.9 (1.6)	92.2 (1.1)	--	d
9	E: lemon soda	No	950, 951, 959	Yes (4.4)	200.4 (0.7)	102.1 (0.3)	--	--
10	A: cola	No	950, 951	No	87.8 (0.5)	307.3 (0.4)	--	--
11	A: cola	No	950, 955	Yes (7)	94.7 (0.3)	--	--	--
12	A: cola	No	950, 951	No	39.3 (0.3)	513.9 (0.4)	--	--
13	H: energetic	No	950, 955, 960	No	165.8 (1.6)	--	--	--
14	B: orange soda	No	950, 951, 954	No	231.7 (0.7)	185.4 (0.3)	37.0 (0.1)	--
15	A: cola	No	950, 951, 952	No	146.8 (0.3)	94.1 (0.6)	--	d
16	E: lemon soda	No	950, 955	Yes (4.7)	66.98 (0.04)	--	--	--

Table S1. Continued

Sample #	Type	Store brand	Sweeteners added (E numbers)	Sugar content (g/100 mL)	Acesulfame K (SD)	Aspartame (SD)	Saccharin (SD)	Cyclamates
17	F: tea-based	No	951, 955	Yes (4.5)	52.8 (0.3)	--	--	--
18	B: orange soda	Yes	950, 951, 955	No	165.7 (0.5)	73.8 (0.3)	--	--
19	E: lemon soda	Yes	950, 951, 952	No	234.84 (0.04)	45.3 (0.5)	--	d
20	G: tonic water	No	952, 954	Yes (2.4)	--	--	57.6 (0.4)	--
21	G: tonic water	Yes	950, 955	Yes (4.8)	23.7 (0.3)	--	--	--
22	G: tonic water	No	**None**	Yes (7.8)	--	--	--	--
23	A: cola	Yes	950, 951	No	145.1 (0.7)	107.1 (0.5)	--	--
24	A: cola	Yes	950, 951	No	167.6 (0.6)	103.6 (0.1)	--	--
25	E: lemon soda	No	950, 955	Yes (4.3)	20.63 (0.08)	--	--	--
26	B: orange soda	No	952, 955	Yes (4.3)	--	--	--	d
27	B: orange soda	No	950, 951, 954	No	227.7 (1.4)	223.2 (0.2)	36.8 (0.2)	--
28	E: lemon soda	No	950, 951, 952	No	226.3 (1.3)	55.9 (0.4)	--	d
29	D: isotonic	No	950, 951, 955	No	70.3 (0.2)	32.6 (0.3)	--	--
30	D: isotonic	No	950, 955	Yes (4.4)	17.92 (0.06)	--	--	--
31	H: energetic	No	**None**	Yes (11)	--	--	--	--
32	G: tonic water	No	950, 952, 954	No	110.0 (1.6)	--	46.0 (0.8)	--
33	B: orange soda	No	950, 952, 955	No	78.7 (0.3)	--	--	--
34	F: tea-based	No	950, 955	Yes (4.5)	48.5 (0.3)	--	--	--
35	A: cola	Yes	950, 952	Yes (4.9)	24.1 (0.1)	--	--	--

Table S1. Continued

Sample #	Type	Store brand	Sweeteners added (E numbers)	Sugar content (g/100 mL)	Acesulfame K (SD)	Aspartame (SD)	Saccharin (SD)	Cyclamates
36	F: tea-based	No	950, 951, 952	No	92.9 (1.2)	58.7 (0.8)	--	d
37	B: orange soda	No	950, 955	No	104.6 (0.6)	--	--	--
38	E: lemon soda	No	950, 951	Yes (4.4)	125.27 (0.02)	98.1 (0.1)	--	--
39	C: “Gaseosa”	Yes	952, 954	No	--	--	77.1 (0.2)	--
40	C: “Gaseosa”	No	952, 954	No	--	--	85.2 (0.5)	--
41	E: lemon soda	No	950, 952, 954	No	72.5 (0.1)	--	51.89 (0.04)	--
42	F: tea-based	No	952, 954, 959	No	--	--	79.9 (0.5)	--
43	A: cola	Yes	950, 951, 952	No	135.4 (0.4)	109.2 (0.2)	--	--
